# Comparative analysis of current 3D printed acetabular titanium implants

**DOI:** 10.1186/s41205-019-0052-0

**Published:** 2019-11-06

**Authors:** Lorenzo Dall’Ava, Harry Hothi, Johann Henckel, Anna Di Laura, Paul Shearing, Alister Hart

**Affiliations:** 1 Institute of Orthopaedics and Musculoskeletal Science, University College London, Brockley Hill, Stanmore, HA7 4LP UK; 2 Royal National Orthopaedic Hospital, Brockley Hill, Stanmore, HA7 4LP UK; 3 Electrochemical Innovation Lab, Department of Chemical Engineering, University College London, Torrington Place, London, WC1E 7JE UK

**Keywords:** 3D printing, Additive manufacturing, Orthopaedic implant, Acetabular cup, Hip Arthroplasty

## Abstract

**Background:**

The design freedom allowed by three-dimensional (3D) printing enables the production of acetabular off-the-shelf cups with complex porous structures. The only studies on these designs are limited to clinical outcomes. Our aim was to analyse and compare the designs of different 3D printed cups from multiple manufacturers (Delta TT, Trident II Tritanium and Mpact 3D Metal).

**Methods:**

We analysed the outer surface of the cups using scanning electron microscopy (SEM) and assessed clinically relevant morphometric features of the lattice structures using micro-computed tomography (micro-CT). Dimensions related to the cup wall (solid, lattice and overall thickness) were also measured. Roundness and roughness of the internal cup surface were analysed with coordinate measuring machine (CMM) and optical profilometry.

**Results:**

SEM showed partially molten titanium beads on all cups, significantly smaller on Trident II (27 μm vs ~ 70 μm, *p* < 0.0001). We found a spread of pore sizes, with median values of 0.521, 0.841 and 1.004 mm for Trident II, Delta TT and Mpact, respectively. Trident II was also significantly less porous (63%, *p* < 0.0001) than the others (Delta TT 72.3%, Mpact 76.4%), and showed the thinnest lattice region of the cup wall (1.038 mm, *p* < 0.0001), while Mpact exhibited the thicker solid region (4.880 mm, *p* < 0.0044). Similar roundness and roughness of the internal cup surfaces were found.

**Conclusion:**

This was the first study to compare the designs of different 3D printed cups. A variability in the morphology of the outer surface of the cups and lattice structures was found. The existence of titanium beads on 3D printed parts is a known by-product of the manufacturing process; however, their prevalence on acetabular cups used in patients is an interesting finding, since these beads may potentially be released in the body.

## Background

More than 90,000 total hip arthroplasties (THA) were performed in the United Kingdom in 2017, with almost 70% using uncemented acetabular cups [1]. Although the majority of these implants were manufactured using traditional technologies [2, 3], around 10% of off-the-shelf designs are now produced using three-dimensional (3D) printing methods [4, 5].

The design freedom allowed by 3D printing enables the production of cups with different features and complex porous structures [6]; the main clinical rationale is to provide enhanced fixation with bone, compared to conventionally manufactured cups [7–9]. This is particularly pertinent given that aseptic loosening is one of the most common reason for revision in THA [1].

Over 60,000 acetabular cups have been produced using electron beam melting (EBM), a common 3D printing technique for metal components [10]. These implants have undergone benchmark testing and clinical evaluation to obtain the required certifications for application [11–13], but the implications of 3D printing manufacturing on the properties and performance of these implants are yet to be published in the literature.

The only published studies to investigate off-the-shelf 3D printed acetabular components have been limited to early to medium term clinical outcomes [14–16] and there have been no studies to report on independent analysis methods for evaluating 3D printed implants.

The aim of this study was to compare different designs of titanium 3D printed off-the-shelf cups for THA. Our first objective was to investigate characteristics of the outer surface of the cups; our second objective was to assess features of the internal cup surface.

## Materials and methods

**Fig. 1:**
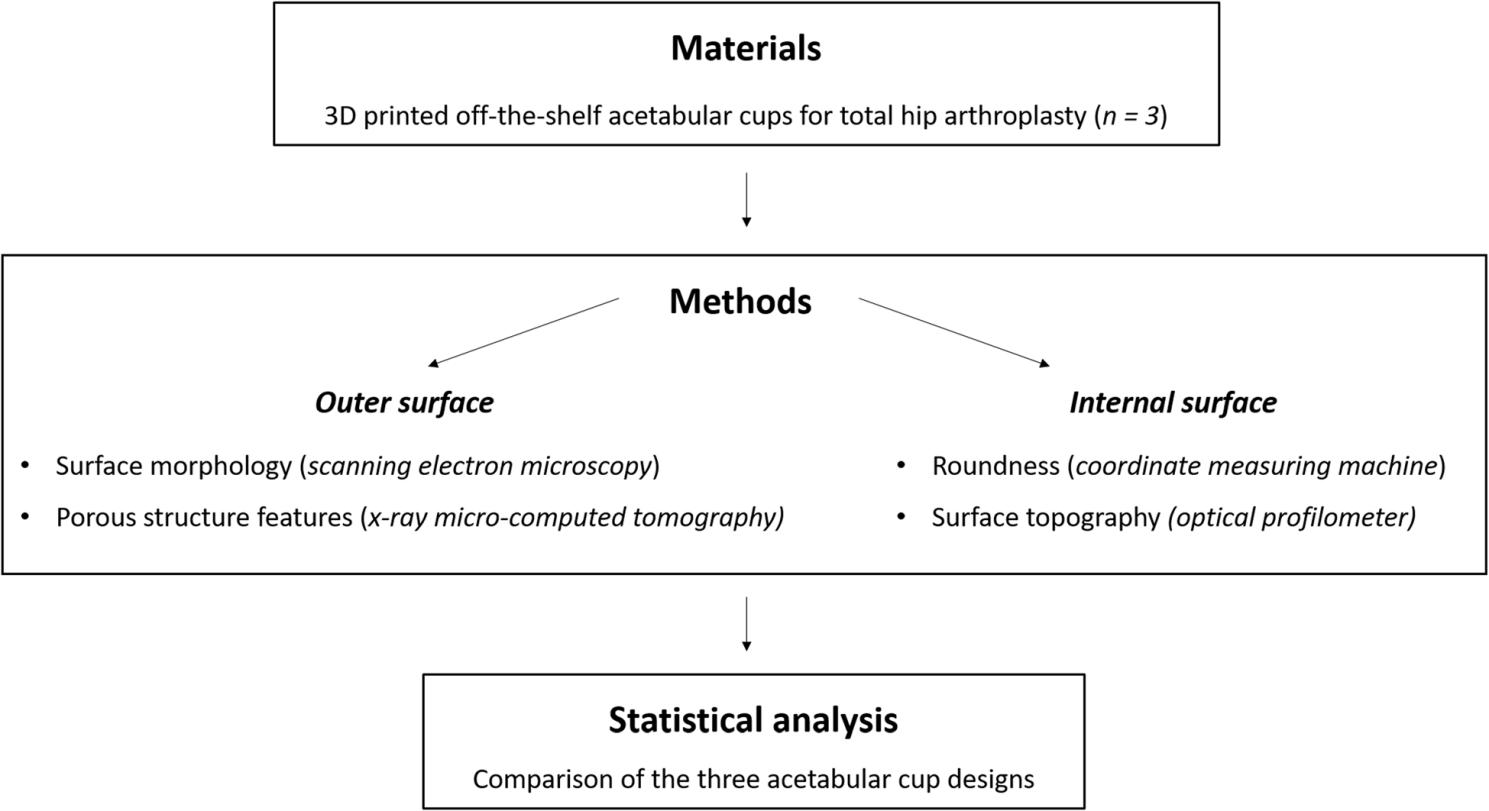
Flow chart of the study design

A schematic flowchart of the study design is shown in Fig. 1.

### Materials

**Fig. 2:**
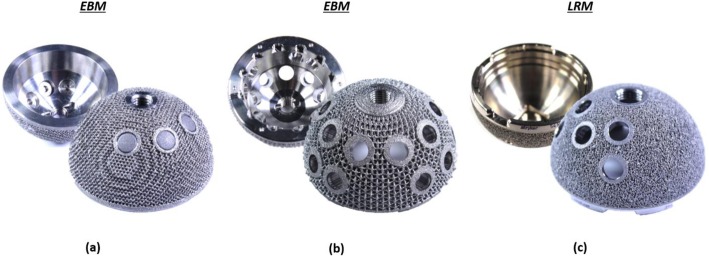
Image showing the internal and the outer surfaces of the three acetabular designs: (**a**) Delta TT (Lima Corporate), (**b**) Mpact 3D Metal (Medacta), (**c**) Trident II Tritanium (Stryker)

This study compared 3 different designs of pristine 3D printed titanium acetabular cups received at our centre: Delta TT (Lima Corporate, Italy), Trident II Tritanium (Stryker, USA), Mpact 3D Metal (Medacta, Switzerland), Fig. 2. All cups were ‘off-the-shelf’ designs (i.e. not patient-matched implants). The Trident II was produced from titanium-aluminium-vanadium alloy (Ti6Al4V) powder using laser rapid manufacturing (LRM) 3D printing [12], was 54 mm in diameter and had 5 screw holes. The Delta TT and Mpact cups were manufactured using electron beam melting (EBM), starting from Ti6Al4V powder [11, 17], were both 58 mm in diameter and had 3 and 17 screw holes, respectively.

### Micro-CT analysis

Micro-computed tomography (micro-CT) was used to characterize the lattice structure on the outer surface of the cups. The analysis involved the following steps: (1) micro-CT data acquisition, (2) data reconstruction and segmentation, (3) measurement of morphometric parameters of the lattice structure and dimensions related to cup wall.

#### Data acquisition

A micro-CT scanner (XTH 225, Nikon Metrology, UK) equipped with a Tungsten target as X-ray source and set at beam voltage and current of 150 kV and 70 μA, respectively, was used to scan the implants. A Cu (0.25 mm) hardware filter was used, located at the beam source, to reduce the beam hardening effect, which involves the attenuation of low energy photons (i.e. soft X-rays) while the X-rays beam moves through the absorbing material. All the scans were performed at a complete 360° rotation at a step size of 0.11°, with 3177 views, 1 frame per view and exposure time of 1000 ms. The images resolution was 33 μm, with a total scan time per sample of 53 min.

#### Data reconstruction and segmentation

A volume model of the scanned implants was reconstructed from the acquired 2D projection images importing the data into CT Pro 3D software (version 4.4, Nikon Metrology, UK), using a filtered back-projection algorithm. During the reconstruction process, a numerical filtering (polynomial correction of second order) was applied to further reduce the beam hardening effect.

The reconstructed images of the implants were segmented and rendered using the 3D micro-CT analysis software Avizo (version 9.0, Thermofisher Scientific, US). Segmentation was performed using a built-in automatic segmentation process based on the “iso-50%” principle, using a specific edge grey value for each implant. The threshold value corresponded with the mid-grey-level between the peaks that coincide with the irradiated materials (in our case Titanium alloy and air as background) in the histogram plot of voxels count versus voxel intensity.

#### Measurements of morphometric parameters

**Fig. 3:**
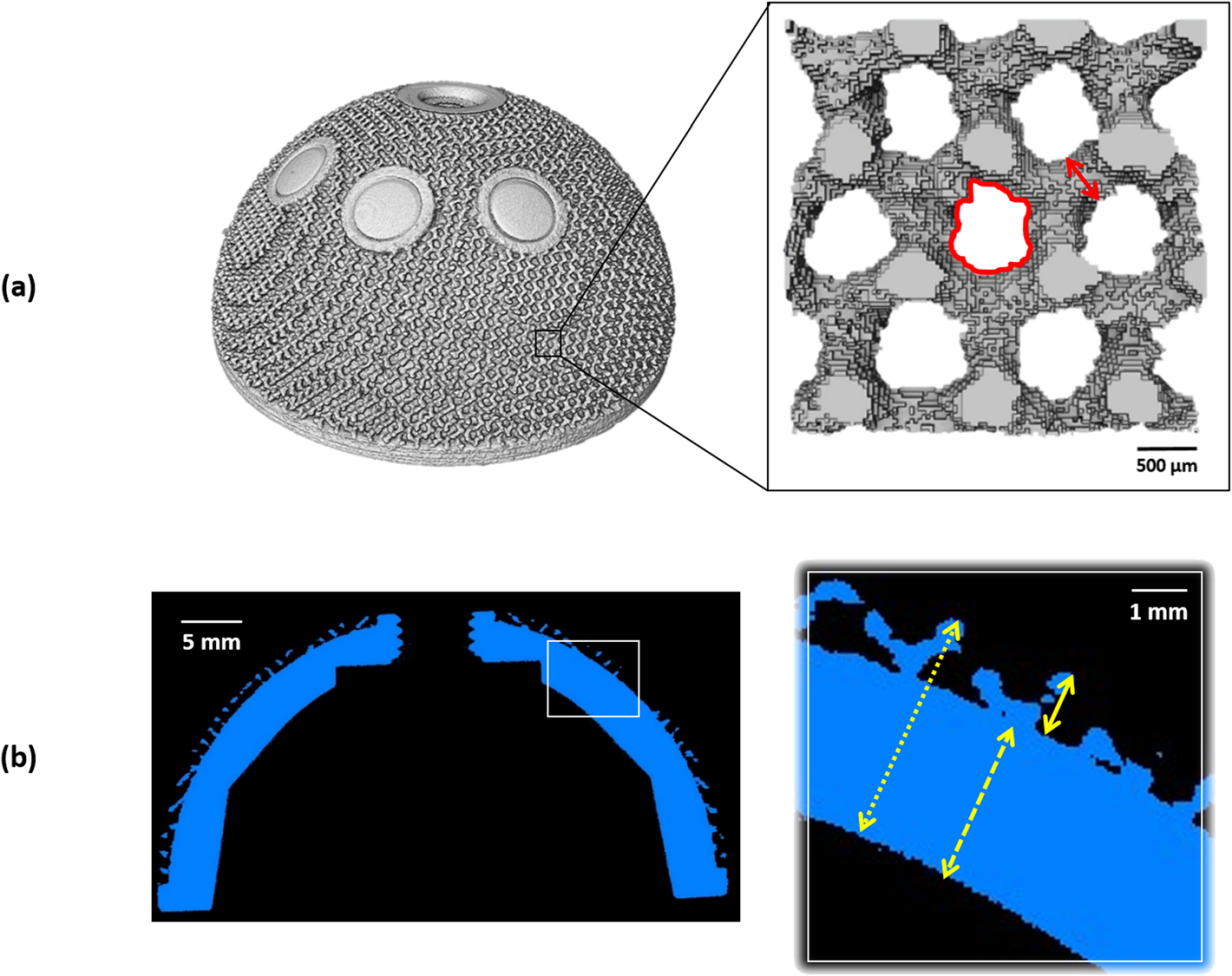
Image summarizing the measured parameters related to the lattice structure and cup wall: (**a**) porosity is the volume of void space in the porous region of interest showed in the zoomed area; pore size is the equivalent diameter of the red shape and strut thickness is indicated by the red arrow; (**b**) lattice thickness, solid thickness and overall thickness of the cup wall are indicated by the solid, dashed and dotted yellow arrows in the zoomed area of the cross-section of the implant, respectively. The volume rendering and the cross-section are of the Delta TT cup

Morphometric features of the lattice structures on the outer surface of the cups were measured: *porosity*, *pore size* and *strut thickness*. The first two parameters represent the percentage of void spaces over the total volume and the equivalent diameter of the pores, respectively; these provide an indication of the available space for bone tissue ingrowth into the porous structure. The third feature is the dimension of the framework of the lattice structure, depicting the available space for bone attachment. Dimensions related to the cup wall were also measured: *solid thickness*, *lattice thickness* and *overall thickness*; the lattice thickness indicates the maximum penetration depth for bone ingrowth (Fig. 3).

All the measurements were performed using Avizo and the public domain software for image analysis ImageJ (version 1.52a, Broken Symmetry Software). Regions of interest confined to the porous network only were selected for porosity, pore size and strut thickness; 15 measurements were made of each parameter on each component. A number of 8 equidistant cross-sections passing for the pole of the cups were selected, taking 5 measurements of solid, lattice and overall thickness on each cross-section for each component.

### Scanning Electron microscopy (SEM)

Detailed analysis of the morphology of the outer surface of the implants was performed using a scanning electron microscopy - SEM (Hitachi S3400-N, Tokyo, Japan). Images were captured in secondary electron imaging (SEI) mode at 20 kV, with magnifications ranging from 20x to 250x.

### Dimensional measurement

The roundness of the internal cup surface was measured using a Zeiss Contura (Carl Zeiss ltd., Rugby, UK) coordinate measuring machine (CMM). Measurements were taken using a 2 mm ruby stylus, recording 5 traces for each component at different heights from the cup rim, with an average of 14,684 points for each trace. Roundness values were automatically computed using the minimum zone circle method (difference between the radius of the most inside and outside points of the profile). The accuracy of the measurement technique is 2.2 + L/350 μm, where L is the measured dimension

### Surface topography analysis

The surface topography of the internal cup surface was investigated using a contour GT-K 3D optical profilometer (Bruker, Coventry, United Kingdom), determining the roughness of the surface. A total of 10 measurements scans were taken, with a scan area of 437.1 μm × 582.7 μm, using a 20x objective lens and 0.55 multiplier. A Gaussian regression filter was applied to the raw data to obtain a measure of Ra roughness

### Statistical analysis

Statistical analysis was performed using the statistical software package Prism (version 7.01, GraphPad, US). The data were assessed for normality using the D’Agostino-Pearson test. Non parametric Kruskal-Wallis and subsequent post hoc Dunn’s multiple comparison tests were used to determine significant differences among the three implants in pore size, lattice and solid thickness of the cup wall, roundness and roughness of the internal cup surface. One-way ANOVA and subsequent post hoc Tukey’s multiple comparison tests were used to determine significant differences among the three cups in porosity, strut thickness and overall thickness of the cup wall. The level of significance for all statistical analyses was *p* < 0.05.

## Results

The results are presented according to the location on the cup, starting with the analysis performed on the outer surface and continuing with the investigation of the internal surface of the three acetabular cup designs.

### Morphometric parameters

Table 1 summarizes the measurements of the morphometric features of the lattice structure for the three implants: porosity, pore size and strut thickness.

**Table 1:** Summary of the median (interquartile range) measurements of the morphometric parameters for the three cups

Characteristic	Delta TT	Mpact	Trident II
Porosity, %	72.3 (70.8 to 74.1)	76.4 (74.9 to 76.7)	63.0 (59.4 to 66.4)
Pore size, mm	0.841 (0.828 to 0.893)	1.004 (0.982 to 1.029)	0.521 (0.371 to 0.648)
Strut thickness, μm	472 (445 to 516)	648 (540 to 675)	421 (326 to 508)

**Fig. 4:**
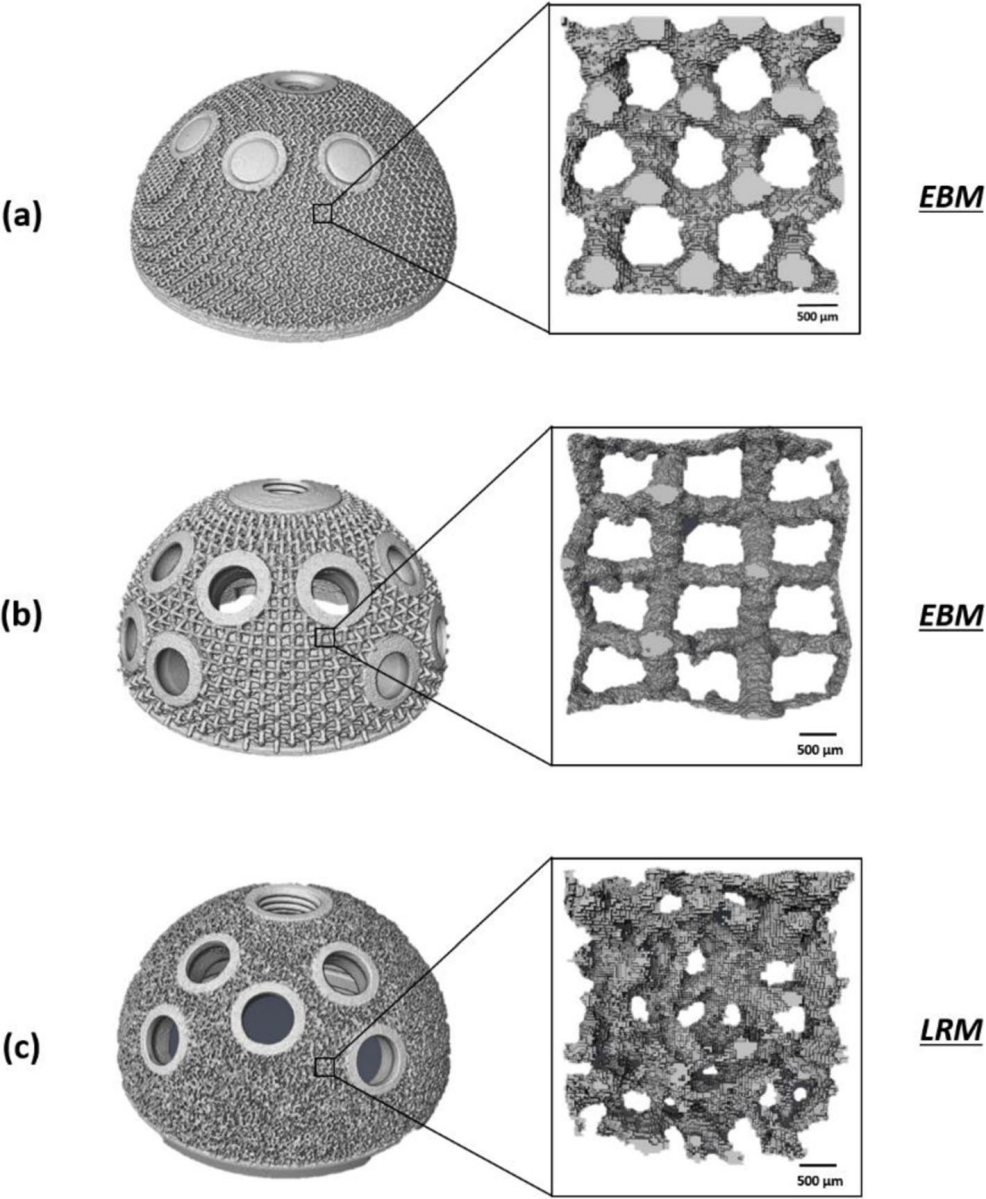
Images showing the micro-CT outcomes for the three cups (**a**) Delta TT, (**b**) Mpact 3D Metal and (**c**) Trident II Tritanium. Different lattice structures with different pore sizes are exhibited

**Fig. 5:**
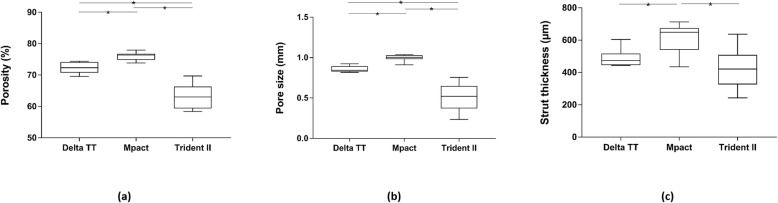
Box plots showing the distribution of the measurements of (**a**) porosity, (**b**) pore size and (**c**) strut thickness of the lattice structure of the three cups. Significant differences (*) were found among the cups

Variability of the features of the lattice structure of the cups was found (Fig. 4). The outer surface of the Trident II design was significantly less porous than Delta TT (*p* < 0.0001) and Mpact (*p* < 0.0001); significant difference was also present between the Delta TT and Mpact cups (*p* = 0.0006). A spread of pore sizes was measured: the Mpact cup showed significantly bigger pores than the Delta TT (*p* = 0.0064) and Trident II cups (*p* < 0.0001); the Trident II cup had also smaller pores than Delta TT (*p* = 0.0048). The struts of the lattice structure were similar between Delta TT and Trident II (*p* = 0.1556), while the Mpact cup showed thicker struts than Delta TT (*p* = 0.0047) and Trident II (*p* < 0.0001). Figure 5 summarizes the measured values in box plot graphs, highlighting the aforementioned differences among the cup designs.

**Fig. 6:**
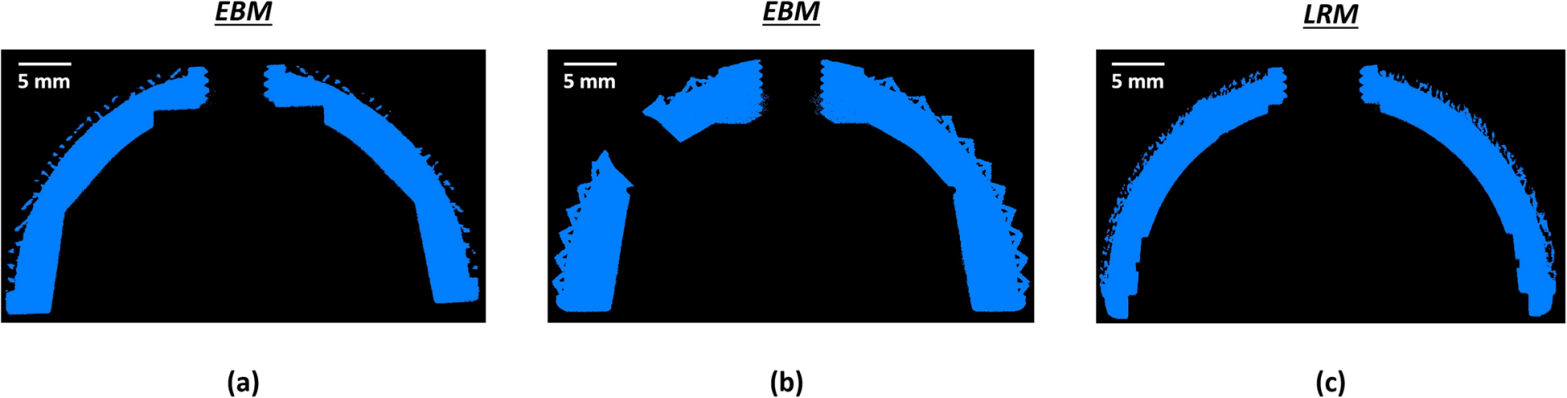
Images showing cross-sections of the three cups (**a**) Delta TT, (**b**) Mpact 3D Metal and (**c**) Trident TT Tritanium. Significant differences were found in the solid, lattice and overall thickness of the cup wall

The values of solid, lattice and overall thickness of the cup wall for the three acetabular components are summarised in Table 2. The Mpact cup showed significantly higher solid thickness than Delta TT (*p* = 0.0044) and Trident II (*p* < 0.0001); Trident II had also a significantly lower solid thickness than Delta TT (*p* < 0.0001). The Trident II cup also exhibited a significantly lower lattice thickness than Delta TT (*p* < 0.0001) and Mpact (*p* < 0.0001), while there was no significant difference between these last two acetabular components (*p* > 0.9999). Significant differences were also present in terms of overall thickness of the cup wall between Mpact and Delta TT (*p* = 0.0064), Mpact and Trident II (*p* < 0.0001) and Delta TT and Trident II (*p* < 0.0001). Overall, the Mpact cup showed the thickest cup wall, both in the solid and lattice regions, with wider distributions of the values compared to the other cups, suggesting less uniformity in the dimensions of the cup wall (Fig. 6).

**Table 2:** Summary of the median (interquartile range) measurements of the cup wall thickness (dense, porous and overall) for the three cups

Characteristic	Delta TT	Mpact	Trident II
Solid thickness, mm	4.626 (4.242 to 4.805)	4.880 (4.774 to 6.095)	3.967 (3.839 to 4.033)
Lattice thickness, mm	1.347 (1.284 to 1.468)	1.468 (1.184 to 1.579)	1.038 (0.966 to 1.085)
Overall thickness, mm	5.973 (5.526 to 6.272)	6.424 (6.099 to 7.442)	4.955 (4.868 to 5.058)

### Scanning Electron microscopy (SEM)

**Fig. 7:**
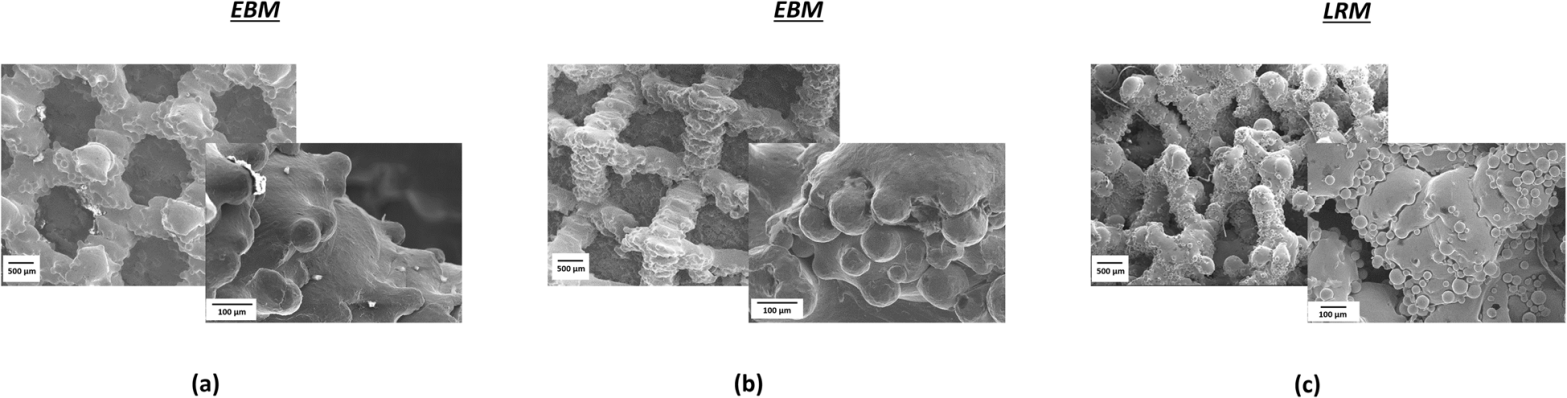
SEM images of the three acetabular designs: (**a**) Delta TT, (**b**) Mpact 3D Metal and (**c**) Trident II Tritanium. At lower magnification (20x), the Trident II design showed an irregular structure, unlike Delta TT and Mpact; at higher magnification (250x), partially molten beads were present on all the cups

The images captured using the SEM for the three acetabular designs are shown in Fig. 7. At low magnification (20x), the Delta TT design exhibited a regular porous structure, where the pores approximately resembled hexagon-shaped unit cells. The Trident II cup showed an irregular porous architecture, with pores of undefined shape. The Mpact design had a regular porous structure with a pyramidal/tetrahedral framework, showing approximately rectangular pores. At higher magnification (250x), all the design exhibited randomly located beads attached to the struts of the porous structure. The beads were present on all the cups, with a significant difference in their dimension between the implants manufactured using EBM (Delta TT, Mpact) and LRM (Trident II) (*p* < 0.0001), but not between the two produced using EBM (*p* = 0.4760). The median (range) size was 0.073 mm (0.055 to 0.100), 0.076 mm (0.041 to 0.093) and 0.027 mm (0.020 to 0.046) for Delta TT, Mpact and Trident II, respectively. The Trident II cup also showed higher density of beads than to the others, with 460 beads/mm^2^, 80 beads/mm^2^ and 50 beads/mm^2^ on average for Trident II, Mpact and Delta TT cup. The layer-over-layer structure typical of 3D printed objects was also visible.

### Dimensional measurements

**Fig. 8:**
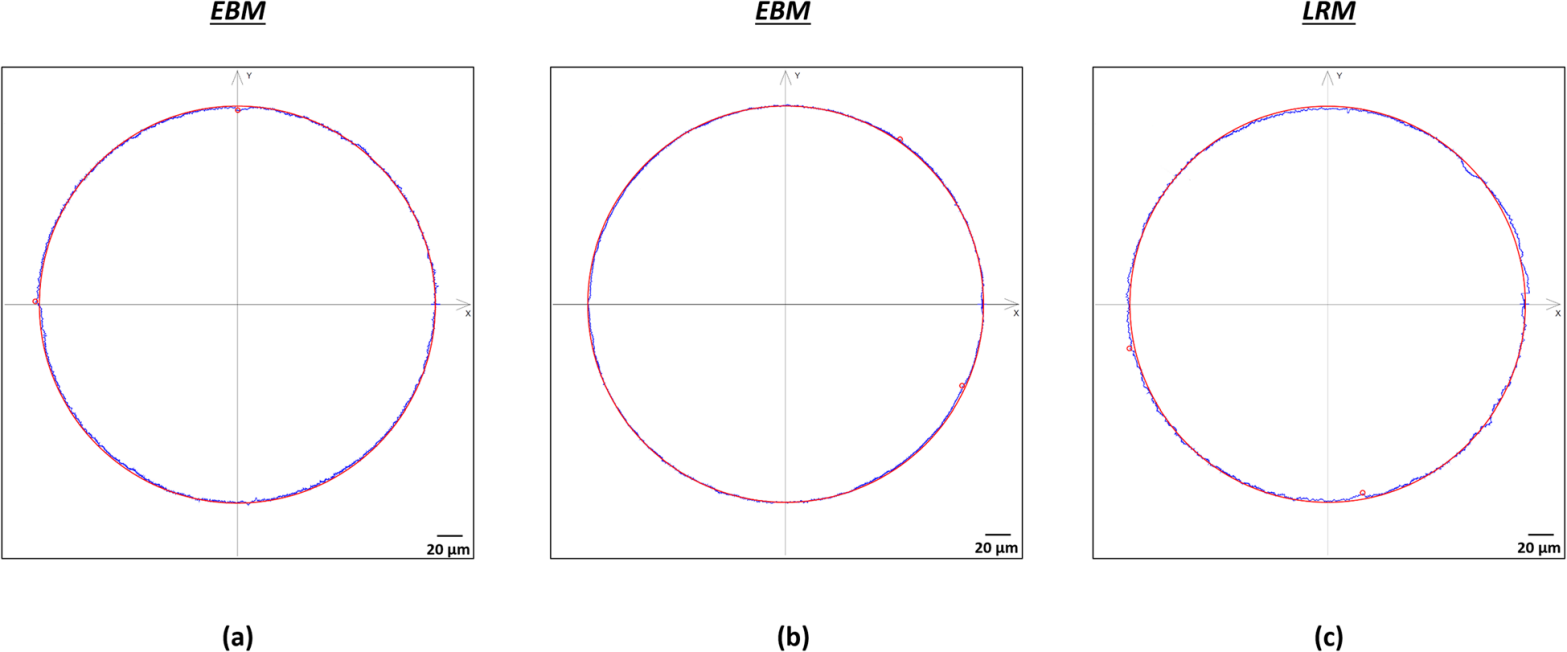
Example of the scans (blue profile) taken using the CMM to measure the roundness of the internal cup surface for the (**a**) Delta TT, (**b**) Mpact 3D Metal and (**c**) Trident II Tritanium cups. The red solid line represents the nominal circle

From CMM analysis of the internal cup surface, a median (IQR) roundness of 8.7 μm (7.4 to 13.95), 6.0 μm (4.15 to 12.4) and 9.5 μm (8.45 to 20.05) were obtained for Delta TT, Trident II and Mpact, respectively. There were no significant differences between the Delta TT and Trident II designs (*p* = 0.8652), Delta TT and Mpact (*p* > 0.9999) and Trident II and Mpact (*p* = 0.1311). Figure 8 shows an example of the scan traces taken using the CMM.

### Surface topography analysis

**Fig. 9:**
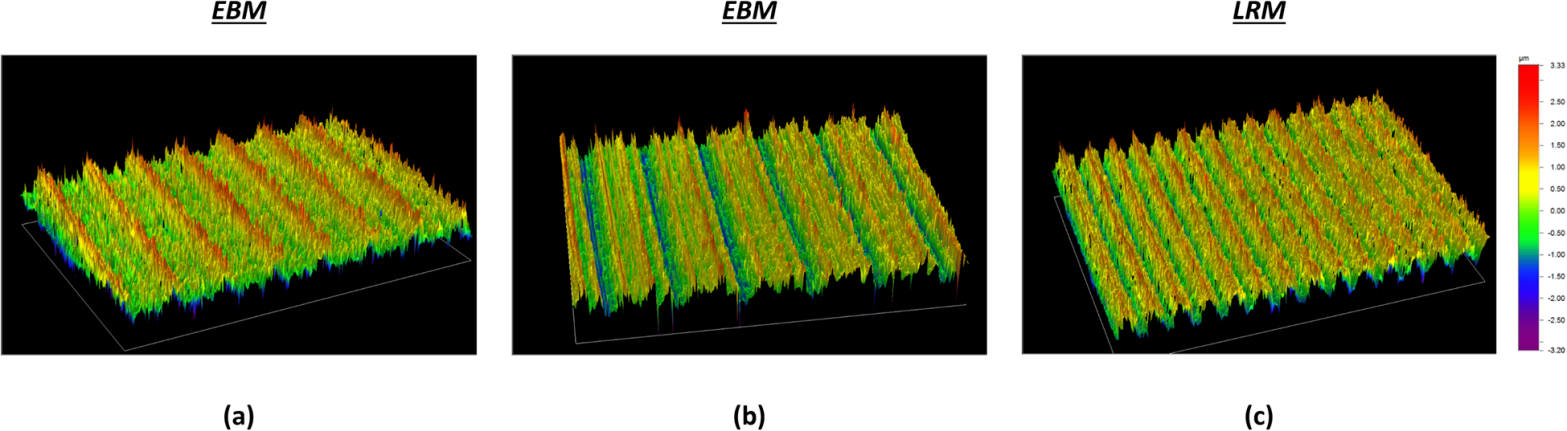
Examples of the scans of the internal cup surface generated by the profilometer for the three acetabular cups: (**a**) Delta TT, (**b**) Mpact 3D Metal and (**c**) Trident II Tritanium. No significant differences in Ra roughness were found among the three designs

The median (IQR) roughness for the Delta TT, Trident II and Mpact cups were 0.549 μm (0.519 to 0.566), 0.552 μm (0.361 to 0.744) and 0.431 μm (0.424 to 0.467), respectively. No significant difference was found between Delta TT and Trident II (*p* > 0.9999), Delta TT and Mpact (*p* = 0.1884) and Trident II and Mpact (*p* = 0.6445) designs. Typical examples of the measurement scans captured with the optical profilometer for the internal cup surface of the three acetabular designs are shown in Fig. 9.

## Discussion

This study is the first to compare different designs of 3D printed off-the-shelf acetabular cups from multiple manufacturers. We found a variability in the morphology of the outer surface due to the different 3D printing process (EBM vs LRM), with evidence of partially molten beads on all the cups. Differences in the lattice structures were also present, with a variability in the pore sizes and porosity of the cups, as well as in the thickness of the cup wall.

The analysis of the morphology of the outer surface of the cups from SEM images revealed the presence of partially molten beads on the struts of the porous structures. Smaller beads and higher beads density (beads/mm^2^) were found on the LRM-manufactured cup (Trident II) compared to the EBM-manufactured cups; this may be due to the smaller titanium powder beads used with the former compared to the latter [6].

These beads are a known by-product of the general 3D printing manufacturing process [18] however the presence of these in final-build acetabular components was noteworthy. The clinical impact of these partially molten beads needs to be understood, particularly if this may increase the release of titanium from the implants. Although titanium has superior biocompatibility, recent studies have revealed a series of adverse effects associated with this metal [19]. To date, one study investigated the possibility of increased systemic titanium level in patients with a 3D printed off-the-shelf cup, finding no differences in comparison with a traditional titanium cup [20]. However, the detection of titanium in blood samples is subjected to interferences from other chemical elements during the analysis; it has been recommended to use a high-resolution induced coupled plasma mass spectroscopy (ICP-MS) to obtain reliable outcomes, detecting titanium more accurately than other instruments [19]. Further studies including 3D printed cups and using ICP-MS might help understand if the presence of the partially molten beads and the higher porosity of these acetabular components may be a concern for the patients. However, it cannot also be excluded that the presence of these beads may promote osseointegration, considering the rough surface that is created.

Three-dimensional printing allows design freedom that conventional manufacturing techniques cannot provide, as shown by the variability described for the three cups analysed using SEM and micro-CT.

The impact of pore size and shape on bone ingrowth and implant osseointegration is still a controversial subject and depends on the geometric and mechanical characteristics of the structure at the bone-implant interface. It has been suggested by in vitro and in vivo studies that pore sizes of both 100–400 μm and 500–1000 μm promote cell growth and proliferation [21–23]. Interestingly, the pores of the cups in this study were not circular in shape, which has been suggested to be more prone to occlusion [23]. Although the specifications of the different pore shapes are proprietary, the methods to define the 3D porous structures are well established [9, 24]. As shown in this study, both regular (repeated unit cells) or irregular (stochastic) structures can be designed and manufactured. Human trabecular bone has an interconnected, open-porous structure with porosity of 50–90%, pore size in the order of 1 mm and trabecular (strut) thickness of hundreds of microns [25, 26]. The highly porous structure of the 3D printed cups showed values similar to those of human bone, with the ability of potentially reduce the risk of stress shielding due to the stiffness mismatch between implant and bone tissue [27, 28]. The hexagon-shaped porous structure, called Trabecular Titanium (TT), showed by the Delta TT cup has been previously characterized using cubic and cylindrical samples [17, 29, 30]. The values of porosity, pore size and strut thickness were comparable to our findings. Similarly, the values of porosity and pore size of the Trident II and Mpact cup were comparable to the specifications provided by the manufacturers [31, 32].

The design freedom of 3D printing enables thinner cup walls to be manufactured for a specific cup diameter. This means that a smaller cup can be chosen for specific head size, therefore sparing more bone stock. The three cups showed different dimensions both in thickness of the cup wall and depth of the porous structure.

The clinical impact of this variability in both the solid and lattice structures of the cups will be better understood from long-term clinical studies involving these 3D printed acetabular components. The Trident II and Mpact cups have recently been introduced on the market. The only study related to the Trident II cup compared the seating and the initial stability of this cup with the Trident I cup, which is conventionally manufactured, in an in vitro bench test using foam blocks [33]. The Delta TT cup has been present since 2007 and clinical studies have reported satisfactory short to mid-term outcomes [14–16, 29].

While differences were found from the analysis of the outer surface of the cups, this was not true for the internal surface. The roundness and roughness of the internal cup surface was measured to identify potential differences in the dimensional and topographic properties of the cups, given by the 3D printing manufacturing method, that could influence the seating of the liner. As previously mentioned, the Trident II cup was produced using LRM, while the Delta TT and Mpact cups using EBM. Despite this difference, and the fact that even the EBM process can have distinct parameter settings between different manufacturers, the three cups showed similar roundness. This can be explained by the fact that 3D printing builds both the solid and lattice structure of the cups in a single stage layer-over-layer, but post-processing such as machining is performed to achieve the required tolerances to avoid dimensional mismatches with the liner and to guarantee its optimal accommodation. If the liner had an incorrect seating, a fracture (if ceramic) or an adverse effect on the fluid-film lubrication may occur, resulting in increased wear [34]. The dimensional tolerance for this feature was not available, however, we found an overall mean roundness of 10.5 μm, suggesting near-perfect round shape of the internal surface of the cups.

No differences were found in the roughness of the internal cup surface. The post-processing of the cups managed to provide a Ra values of around 0.5 μm; it has been suggested that 3D printing parts for high end applications, such as orthopaedic implants, should have a surface roughness of less than 1 μm [18]. An elevated surface roughness might influence mechanical wear and corrosion between the cup and the liner, because the reduced contact area between the two surfaces (internal of the cup, backside of the liner) might lead to increased localized stresses and more space for fluid ingress.

We acknowledge as limitations of this study the small cohort of acetabular cups under analysis and the non-uniformity in the cup size. The analysis of orthopaedic implants, especially if recently marketed, can be difficult. Further studies including more implants are needed to better understand the impact of the differences that we described.

## Conclusion

This was the first study to compare different designs of 3D printed off-the-shelf acetabular cups from multiple manufacturers. We found a variability in the morphology of the outer surface of the cups and the properties of the porous structure. Although the existence of partially molten surface beads on 3D printed parts is a known by-product, their prevalence on these acetabular cups was interesting and the related clinical implications, if any, need to be investigated.

This comparison of different designs of 3D printed cups provides manufacturers and regulators, such as the Food and Drug Administration (FDA), the Medicine and Healthcare Products Regulatory Agency (MHRA) and the British Standards Institution (BSI) with evidence that may help to build robust investigation methods for this type of components and to monitor the implants that are already present on the market. Further laboratory studies, analysis of retrieved components and long-term clinical outcomes will help to prevent another metal-on-metal experience from happening.

## Data Availability

The datasets used and/or analysed during the current study are available from the corresponding author on reasonable request.
